# Cellular heterogeneity in TNF/TNFR1 signalling: live cell imaging of cell fate decisions in single cells

**DOI:** 10.1038/s41419-024-06559-z

**Published:** 2024-03-11

**Authors:** Marcus K. Preedy, Michael R. H. White, Vinay Tergaonkar

**Affiliations:** 1 Laboratory of NF-κB Signalling, Institute of Molecular and Cell Biology (IMCB), Agency for Science, Technology and Research (A*STAR), 61 Biopolis Drive, Proteos, Singapore, 138673 Singapore; 2 Division of Molecular and Cellular Function, School of Biological Sciences, Faculty of Biology, Medicine and Health, The University of Manchester, Michael Smith Building, D3308, Dover Street, Manchester, M13 9PT England UK; 3 Department of Biochemistry, Yong Loo Lin School of Medicine, National University of Singapore (NUS), 8 Medical Drive, MD7, Singapore, 117596 Singapore

**Keywords:** Tumour heterogeneity, Apoptosis, Necroptosis, Epigenetics, Cell signalling

## Abstract

Cellular responses to TNF are inherently heterogeneous within an isogenic cell population and across different cell types. TNF promotes cell survival by activating pro-inflammatory NF-κB and MAPK signalling pathways but may also trigger apoptosis and necroptosis. Following TNF stimulation, the fate of individual cells is governed by the balance of pro-survival and pro-apoptotic signalling pathways. To elucidate the molecular mechanisms driving heterogenous responses to TNF, quantifying TNF/TNFR1 signalling at the single-cell level is crucial. Fluorescence live-cell imaging techniques offer real-time, dynamic insights into molecular processes in single cells, allowing for detection of rapid and transient changes, as well as identification of subpopulations, that are likely to be missed with traditional endpoint assays. Whilst fluorescence live-cell imaging has been employed extensively to investigate TNF-induced inflammation and TNF-induced cell death, it has been underutilised in studying the role of TNF/TNFR1 signalling pathway crosstalk in guiding cell-fate decisions in single cells. Here, we outline the various opportunities for pathway crosstalk during TNF/TNFR1 signalling and how these interactions may govern heterogenous responses to TNF. We also advocate for the use of live-cell imaging techniques to elucidate the molecular processes driving cell-to-cell variability in single cells. Understanding and overcoming cellular heterogeneity in response to TNF and modulators of the TNF/TNFR1 signalling pathway could lead to the development of targeted therapies for various diseases associated with aberrant TNF/TNFR1 signalling, such as rheumatoid arthritis, metabolic syndrome, and cancer.

## Facts

- TNF is a pro-inflammatory cytokine that is secreted by immune cells in response to harmful stimuli.
- Stimulation with TNF promotes cell survival by activating pro-inflammatory signalling pathways but may also trigger apoptosis and necroptosis.
- Cellular responses to TNF exhibit significant heterogeneity, both within an isogenic cell population and across different cell types.
- Cell-to-cell variability in response to TNF poses a significant challenge to the successful treatment of cancer.

## Questions

- What are the mechanisms driving heterogenous responses to TNF?
- How does signalling pathway crosstalk guide cell-fate decisions in response to TNF?
- Can fluorescence live-cell imaging be utilised to investigate TNF/TNFR1 signalling pathway heterogeneity at the single-cell level?
- Could sensitising resistant cancer cells to TNF-induced cell death be an effective strategy for the treatment of cancer?

## Introduction

Tumour Necrosis Factor alpha (TNF) is a pro-inflammatory cytokine that plays a central role in regulating innate immune and inflammatory responses. Stimulation of cells with TNF activates a series of complex signalling cascades that drive cell-fate decisions. Heterogeneity within the TNF signalling network can be observed in the dynamics of receptor binding, signalling pathway interactions and gene expression. Whilst each of these signalling events is transient, precise regulation is required as the resulting cell-fate decision is often irreversible. The most fundamental decision is binary: should a cell survive or commit to apoptosis? In the normal life of an organism, it is advantageous for apoptotic decision-making to be heterogenous and dynamic, so that not all cells die at the same time. If a population of cells is exposed to harmful stimuli, damaged cells will be removed by apoptosis. However, the innate heterogeneity in pro-apoptotic signalling creates the probability of a surviving subpopulation (known as fractional killing). The mechanisms that create this natural heterogeneity pose a challenge when the objective is to eliminate an entire cell population, such as in the treatment of cancer. This review focuses on the application of fluorescence live-cell imaging to analyse cellular heterogeneity in TNF/TNFR1 signalling and its influence on cell-fate decisions.

TNF controls inflammatory signalling and cell fate through binding to two distinct receptors: TNFR1 and TNFR2, which are differentially expressed in different cell types. TNFR1 contains a death domain (DD) in its cytoplasmic tail, whilst TNFR2 lacks a DD [1, 2]. The single-cell response to TNF stimulation is largely controlled by the balance of TNFR1 and TNFR2 expression. TNFR1 is broadly expressed, and signalling through this receptor will be the focus of this review. TNFR2 expression in contrast is limited to specific cell types, creating natural cell-type heterogeneity at the level of TNF-receptor binding [2–5]. TNF/TNFR2 recruits downstream signalling components independently of DD interactions and is associated with immune modulation and tissue homoeostasis [2, 6]. TNFR2 is overexpressed in many cancers [7], where TNFR2-expressing cells can recruit and activate immunosuppressive cells to support immune escape and tumour development [8]. Targeting TNF/TNFR2 signalling is therefore a promising candidate for cancer immunotherapy [6, 9].

TNF/TNFR1 signalling is facilitated by context-dependent homomeric DD interactions between TNFR1 trimers and downstream signalling components. These differential and dynamic signalling interactions define a second level of heterogeneity in the response to TNF. TNF/TNFR1 signalling can induce the formation of at least three distinct signalling complexes in a context-dependent manner. Complex I formation leads to activation of NF-κB and MAPK signalling and is associated with inflammation and cell survival [10]. Alternatively, complex IIa and IIb both lead to cell death by inducing apoptosis and necroptosis, respectively [11]. TNF/TNFR1 signalling through complex I coordinates immune and inflammatory responses by promoting transcriptional upregulation and secretion of various cytokines and inflammatory mediators [12–14]. Signalling through complex II helps to maintain tissue homoeostasis by eliminating damaged or infected cells [15]. The delicate balance between these two signalling pathways plays a pivotal role in determining cell fate, regulating inflammation, and preserving immune system integrity.

Heterogeneity in TNF/TNFR1 signalling can be observed throughout different stages of the signalling network. Stochastic variation in early events such as TNF-TNFR1 binding controls downstream signalling pathway activation [16, 17]. The dynamic nature of these signalling cascades leads to a further level of cell-to-cell heterogeneity. The key transcription factor NF-κB translocates to the nucleus in response to TNF. In some cells, this is a single cycle of nuclear translocation. In other cells, delayed negative feedback loops in the NF-κB system, most importantly via IκBα and A20 [18–22], drive regular cycles of NF-κB translocation into and out of the nucleus [23]. The activation dynamics of NF-κB [24–27] determine the specific gene expression profile of individual cells [23, 28–30]. Precise integration of dynamic signalling events and downstream gene expression is therefore important for determining the appropriate fate of a cell in response to TNF.

Given that TNF/TNFR1 signalling interactions are transient and dynamic, they need to be measured quantitatively and dynamically in real-time at the single-cell level to elucidate the molecular mechanisms driving heterogenous TNF responses and ultimate cell-fate decisions. Live-cell imaging has been the technique of choice to study these processes. Whilst this approach has been employed extensively to investigate TNF-induced inflammation [18, 23, 31–33], and to some extent TNF-induced cell death [34–36], the role of TNF/TNFR1 signalling pathway crosstalk in guiding cell-fate decisions in single cells has been understudied. It is vital to understand the mechanisms driving cell-to-cell variability in response to TNF and modulators of the TNF/TNFR1 signalling pathway, as drugs designed to promote TNF-induced cell death suffer from fractional killing [37–40], and poor responses in various cell lines [39, 41].

Abnormal TNF/TNFR1 signalling is associated with a wide range of human ailments, spanning from rheumatoid arthritis [42, 43] and metabolic syndrome [44] to cancers [45–47]. Several of these diseases are associated with chronic inflammation, as elevated levels of TNF drive TNF/TNFR1 signalling through complex I [43, 47, 48]. TNF production is itself upregulated by TNF/TNFR1 signalling, establishing a positive feedback loop that amplifies inflammation. Pulsatile and localised TNF secretion is therefore a further driver of cell and tissue heterogeneity that can direct discrete patterns of NF-κB dynamics and gene expression [31, 33]. Anti-TNF therapy is an effective treatment for chronic inflammatory diseases [49–51]. However, long-term TNF blockade can give rise to significant side-effects due to immune suppression, such as opportunistic and viral infections [52, 53]. TNFR1-deficient mice are highly susceptible to infection by Gram-positive bacteria [54, 55] and viruses [56, 57]. TNFR1 knock-out (KO) mice are also resistant to TNF injection [58], which induces lethal septic shock in wild type mice through RIPK1 kinase activity-dependent cell death [59]. These findings demonstrate the pleiotropic effects of TNF and suggest that its complete blockade may not be a suitable long-term treatment for chronic inflammatory diseases.

There is strong evidence to suggest that dysregulation of complex II signalling, leading to erroneous cell death, is a contributor to pathology in chronic inflammation, autoimmune diseases, and viral infection [14, 60–62]. In inflammatory bowel disease, increased TNF-induced cell death can lead to disintegration of the epithelial barrier and subsequent bacterial infiltration. This drives intestinal inflammation, as observed in Crohn’s disease and ulcerative colitis [63]. Establishing a comprehensive understanding of the interplay between TNF/TNFR1 complex I and complex II signalling in single cells will therefore be important to elucidate the underlying mechanisms of diseases associated with aberrant TNF/TNFR1 signalling. Specific non-coding RNAs [64, 65] and short peptides [66, 67] have been identified as key regulators of inflammatory and immune responses. These intracellular components represent potential targets for small molecules and could therefore enable the development of “precision” TNF drugs that only target specific signalling arms, as opposed to the entire signalling cascade.

### TNF/TNFR1 signalling through complex I

**Fig. 1:**
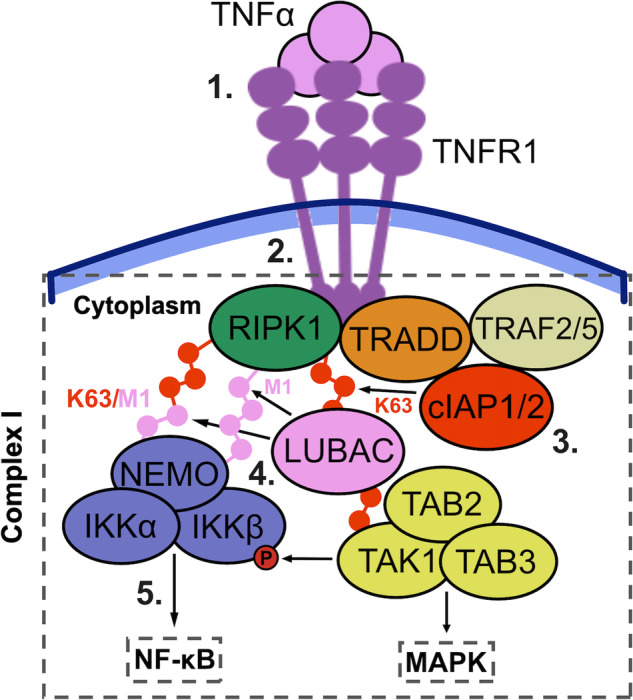
TNF/TNFR1 signalling through complex I. **1** Trimeric TNF binds to TNFR1 on the cell-surface membrane of target cells and induces oligomerization of the receptor. **2** TRADD and RIPK1 are recruited to the intracellular domains of TNF/TNFR1 through their ‘death domain’. These proteins then recruit TRAF2/5 and cIAP1/2 to form TNF/TNFR1 complex I. **3** cIAP1/2 adds K63-linked ubiquitin chains to RIPK1, allowing for the recruitment of LUBAC and TAB2/3. TAB2/3 recruits TAB1 and TAK1. TAK1 then activates the MAPK signalling pathway. **4** LUBAC adds M1-linked ubiquitin chains to RIPK1 and potentially generates K63/M1-linked hybrids. M1- and K63/M1- linked ubiquitin chains on RIPK1 allow for the recruitment of the IKK complex through NEMO. Recruitment of the IKK complex to TNFR1 brings it in proximity to TAK1, which phosphorylates and activates IKK2. **5** IKK2 phosphorylates IκBα, leading to its ubiquitin-mediated proteasomal degradation and liberation of NF-κB, thus activating the NF-κB signalling pathway.

Upon TNF binding, TNFR1 undergoes receptor trimerization. This brings together the cytoplasmic domains of TNFR1, allowing interaction with various adaptor and signalling proteins. TNFR1 recruits TRADD and RIPK1 through homomeric DD interactions [68, 69]. TRADD acts as a scaffold for the recruitment of TRAF2/TRAF5 and cIAP1/cIAP2 to form TNF/TNFR1 complex I [68, 69] (Fig. 1). cIAP1/cIAP2 are E3 ubiquitin ligases that catalyse the formation of K63-linked ubiquitin chains on TNF/TNFR1 complex I components, including RIPK1 [70–73]. Ubiquitin-modified RIPK1 enables the recruitment of TAB2 and TAB3 to the complex [74–77], which in turn recruit TAB1 and TAK1 [78, 79]. TAK1 is a serine/threonine protein kinase that activates the MAPK pathway [80, 81]. Ubiquitin-modified RIPK1 recruits LUBAC, a heterotrimeric complex composed of HOIL-1L, HOIP, and SHARPIN [82–85]. LUBAC is an E3 ubiquitin ligase that conjugates complex I components, including RIPK1, with linear M1-linked ubiquitin and can potentially generate hybrid K63/M1-linked chains [85–88].

The IKK complex, consisting of IKK1, IKK2, and NEMO [89–91], is recruited to TNF/TNFR1 complex I through the binding of NEMO to ubiquitinated RIPK1 [76, 92]. NEMO binds to K63-linked [76, 93, 94], M1-linked [95, 96], and hybrid K63/M1-linked ubiquitin chains [87, 88]. Recruitment of NEMO to TNF/TNFR1 complex I brings the IKK complex in proximity to TAK1, which phosphorylates and activates IKK2 [92]. Active IKK2 phosphorylates IκBα, leading to its ubiquitin-mediated proteasomal degradation. This liberates NF-κB from its cytoplasmic inhibitory complex, allowing its translocation to the nucleus, where it induces the expression of inflammatory and anti-apoptotic genes [30]. NF-κB, as described above, induces the expression of negative regulators of its own signalling pathway, including IκBα, IκBβ, IκBε, and A20 [18–22]. These delayed negative feedback mechanisms lead to oscillatory NF-κB dynamics and in other contexts ensure the appropriate cessation of pro-inflammatory signalling upon removal of harmful stimuli, thus promoting the restoration of cellular homoeostasis.

### TNF/TNFR1 signalling through complex II

**Fig. 2:**
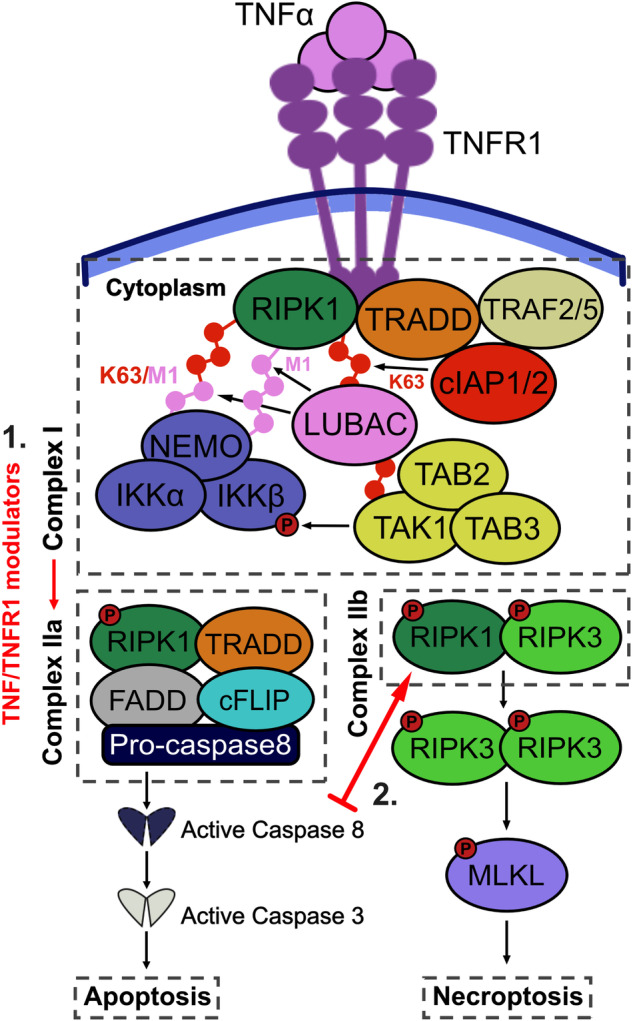
TNF/TNFR1 signalling through complex II. TNF stimulation predominantly induces the formation of TNF/TNFR1 complex I, leading to activation of pro-inflammatory NF-κB and MAPK signalling pathways. However, TNF/TNFR1 signalling may also trigger apoptosis and necroptosis via complex IIa and IIb, respectively. **1** TNF/TNFR1-disrupting agents, such as SMAC mimetics and TAK1 inhibitors, can promote the dissociation of complex I and formation of complex II. In complex IIa, RIPK1 associates with TRADD, FADD, cFLIP, and pro-caspase 8. The high local concentration of pro-caspase 8 induces caspase 8 activation through autocleavage and trans-cleavage from other active caspases. Caspase 8 then cleaves and activates caspase 3, which in turn cleaves downstream components to induce apoptosis. **2** If caspase activation is inhibited, complex IIb can prevail as the dominant signalling pathway. In this pathway, RIPK1 is phosphorylated by RIPK3, causing it to dissociate from complex IIa and instead form a RIPK1-RIPK3 pro-necrotic complex. RIPK3 then directly phosphorylates MLKL, causing it to oligomerise and translocate to the plasma membrane. MLKL binds to phosphatidylinositol phosphates on the membrane’s inner leaflet and disrupts the integrity of the cell membrane, resulting in the release of intracellular contents. This process leads to cell swelling, rupture, and ultimately culminates in cell death by necroptosis.

The post-translational modification (PTM) profile of RIPK1 is believed to play a pivotal role in determining whether propagation of TNF/TNFR1 signalling primarily occurs through complex I or complex II [97–99]. In addition to ubiquitination, RIPK1 is subject to phosphorylation by various kinases, including IKK2 [100], MK2 [101–103], TBK1, and IKKε [104], among others. These phosphorylation events have been shown to protect against RIPK1 kinase-dependent cell death, either by repressing RIPK1 kinase activity or inhibiting the binding of RIPK1 to complex II components, such as FADD and caspase 8. Notably, both IKK2 and MK2 also mediate the activation of further signalling events downstream of complex I, leading to the expression of TNF-induced pro-inflammatory and anti-apoptotic genes [30, 105]. This suggests that complex I formation not only inhibits complex II activation via the induction of anti-apoptotic genes but also through the post-translational regulation of RIPK1. Under specific conditions (discussed in the next section), the PTM profile of RIPK1 can promote its dissociation from complex I to form complex IIa (Fig. 2). Auto-phosphorylation of RIPK1 at serine 166 [106] is thought to be a key driver of this switch.

In complex IIa, RIPK1 associates with TRADD, FADD, pro-caspase 8, and c-FLIP. The high local concentration of complex IIa components leads to proximity activation of caspase 8 via pro-caspase 8 auto-cleavage and trans-cleavage from other active caspases. Caspase 8 then cleaves and activates executioner caspases, such as caspase 3, which cleave downstream components including ICAD, iPLA2, and XKR8 to induce apoptosis [107–109]. Interestingly, caspase 8 has also been shown to directly cleave RIPK1 [110, 111]. RIPK1 cleavage has been suggested to promote TNF-induced apoptosis by inhibiting NF-κB and the expression of pro-survival genes [110, 112]. However, other recent studies have suggested that caspase 8-mediated RIPK1 cleavage may be important for limiting apoptotic and necroptotic forms of cell death [113, 114]. Highlighting the significance of RIPK1 cleavage in protecting against cell death-driven inflammation, Lalaoui et al. [115] and Tao et al. [116] have identified specific monogenic single amino acid mutations in humans that render RIPK1 uncleavable, leading to the early onset of autoinflammatory disease.

When complex IIa forms but caspases are not activated, complex IIb can prevail as the dominant signalling pathway [117–119]. In these circumstances, RIPK1 is phosphorylated by RIPK3, causing dissociation from complex IIa and formation of a RIPK1-RIPK3 pro-necroptotic complex [120–122]. The formation of RIPK1-RIPK3 heterodimers also promotes RIPK3 homodimerization [123]. RIPK3 homodimers are required for necroptosis and are sufficient to induce MLKL-dependent cell death [123]. Active RIPK3 directly phosphorylates MLKL at threonine 357 and serine 358 within its activation loop, triggering a conformational change that induces the formation of higher-order MLKL oligomers [124–127]. These oligomers translocate to the plasma membrane, where they bind to phosphatidylinositol phosphates on the membrane’s inner leaflet. Membrane-bound MLKL oligomers disrupt the integrity of the cell membrane, resulting in the release of intracellular contents. This process leads to cell swelling, rupture, and ultimately culminates in cell death by necroptosis [128].

Interestingly, genetic deletion of RIPK1 has been shown to promote TNF-induced necroptosis via RIPK3 [129, 130]. Wang et al. [131] demonstrated that in RIPK1 KO cells, TRADD forms a complex with RIPK3, which promotes RIPK3 oligomerization and phosphorylation, leading to activation of MLKL and subsequent necroptosis. This implies a level of redundancy between TNF/TNFR1 complex II components, as TRADD can replace the role of RIPK1 during complex IIb-mediated signalling in RIPK1 KO cells. This redundancy is important in development, as ablation of TRADD can rescue *Ripk1*^*−/−*^ *Ripk3*^*−/−*^ mice from perinatal lethality [132, 133]. Whilst deletion of TRADD fails to rescue the survival of *Ripk1*^*−/−*^ mice, it is sufficient to reduce systemic cell death and inflammation in *Ripk1*^*−/−*^ neonates [132]. Interestingly, TRADD is essential for TNF-induced NF-κB activation in *Ripk1*^*−/−*^ *Ripk3*^*−/−*^ fibroblasts [133], suggesting that it plays an important redundant role in both TNF/TNFR1 complex I- and complex II-mediated signalling.

### The interplay between TNF/TNFR1 complex I and complex II signalling is a key determinant in regulating cell fate

Initial investigations into TNF-induced apoptosis demonstrated that activation of NF-κB by TNF protects against cell death [134–138]. Multiple studies have demonstrated that NF-κB activates a set of genes that cooperatively suppress TNF-induced apoptosis [139–141]. These target genes include cIAP1, cIAP2, and XIAP [142]. cIAP1/2 limits extrinsic apoptosis by preventing caspase 8 activation and weakly inhibiting executioner caspases [143–145]. cIAP1/2 are also required for proper activation of the NF-κB signalling pathway [144, 145]. XIAP suppresses apoptosis by inhibiting initiator and executioner caspases [146, 147]. Anti-apoptotic members of the BCL-2 family of proteins have also been identified as NF-κB transcriptional targets [148, 149]. These proteins bind to the outer mitochondrial membrane to prevent mitochondrial outer membrane permeabilization and thus directly inhibit intrinsic apoptosis.

Two separate studies demonstrated that c-FLIP, a negative regulator of apoptosis, is induced by NF-κB [150, 151]. Micheau and Tschopp [138] reported that treatment of cells with TNF induces the sequential formation of TNF/TNFR1 complex I and complex II. The authors proposed that NF-κB signalling downstream of complex I promotes the upregulation of c-FLIP, which inhibits caspase 8 and thus hampers the apoptotic function of complex II. However, if NF-κB activation is defective, c-FLIP expression will not surpass the threshold required to protect cells from TNF-induced apoptosis. TNFR1-mediated signal transduction therefore includes a checkpoint, resulting in cell death (via complex II) in instances where the initial signal (via complex I, NF-κB) fails to activate appropriately. There is a clear evolutionary rationale for the existence of this checkpoint, given that pathogens have developed mechanisms to disrupt TNF/TNFR1 complex I signalling as a protective measure against the host’s immune response [152–154]. If TNF/TNFR1 signalling is activated in response to infection, complex I-mediated inflammatory signalling will be engaged. If the invading pathogen significantly disrupts this pathway, and thus curtails the expression of pro-survival genes, subsequent complex II-mediated signalling will promote cell death to eradicate the pathogen. The dynamic interplay between hosts and pathogens can be viewed as a biological conflict system [155]. Such systems give rise to evolutionary arms races, wherein hosts face selective pressure to evolve resistance to pathogens, whilst pathogens simultaneously strive to develop countermeasures to evade host surveillance and establish a successful infection [156].

Several TNF/TNFR1 components play a role in both complex I- and complex II-mediated signalling pathways. As previously mentioned, RIPK1 is a core component of pro-inflammatory, pro-apoptotic and pro-necroptotic complexes [157]. A20/*TNFAIP3* has been implicated as an important negative regulator of both complex I-induced inflammation and complex II-induced cell death. A20/*TNFAIP3* is a TNF-inducible dual ubiquitin-editing enzyme [158]. It is one of the key negative feedback loops that regulate the dynamics and function of the NF-κB signalling system and has been implicated in controlling the repeated response to pulsatile TNF signalling [33]. It has also been proposed to regulate the timing of NF-κB oscillations through both the level of heterogeneous A20 expression and as a sensor of temperature [159]. The functional importance of A20/*TNFAIP3* was indicated by the observation that A20-deficient mice develop severe inflammation and cachexia, are hypersensitive to TNF, and die prematurely [160]. A20 has unique properties as a ubiquitin-modifying enzyme, displaying deubiquitinating (DUB), E3 ubiquitin ligase, and ubiquitin-binding activities [161]. ZnF4, the domain of A20 with E3 ubiquitin ligase activity, has been shown to bind to K63-linked ubiquitin chains on complex I components [162]. This is suggested to aid recruitment of A20 to the receptor complex and protect K63-linked ubiquitin chains from degradation [96]. Transgenic mice with inactivating mutations in either A20’s DUB [163–165] or ZnF4 domains [163, 165] are grossly normal and do not develop the severe phenotype of A20-deficient mice.

**Fig. 3:**
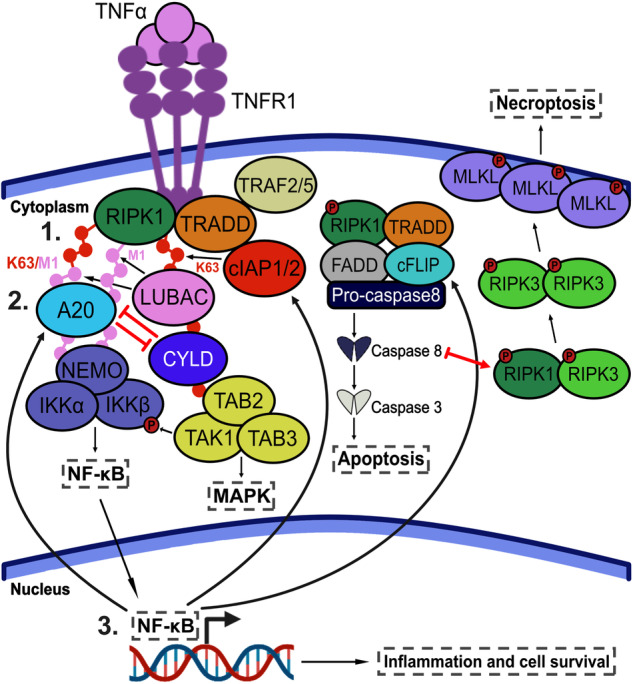
Interplay between TNF/TNFR1 complex I and complex II signalling pathways. TNF stimulation activates a series of complex signalling cascades that drive cell-fate decisions. The intricate interplay between these signalling pathways plays a pivotal role in determining a cell’s response to TNF. There are several checkpoints throughout TNF/TNFR1 signalling where crosstalk can occur. **1** One of the primary components believed to govern the interplay between TNF/TNFR1 complex I- and complex II-mediated signalling occurs during the early stages of complex formation. The post-translational modification (PTM) profile of RIPK1 determines whether signalling will predominantly propagate from complex I or complex II [97]. Under specific conditions, RIPK1 can experience significant PTM alterations, causing it to dissociate from TNF/TNFR1 complex I and instead form complex IIa. **2** The PTM profile of RIPK1 is thought to be regulated by the antagonistic interactions of A20 and CYLD. A20 binds to M1-linked chains on RIPK1 and protects them from degradation by CYLD, thus stabilising complex I. If CYLD successfully removes M1-linked ubiquitin, RIPK1 is more likely to dissociate from complex I and form complex IIa. **3** TNF/TNFR1 complex I-driven activation of NF-κB leads to increased expression of pro-survival genes, such as cIAP1/2 and c-FLIP. c-FLIP directly inhibits activation of pro-caspase 8, thus reducing signalling downstream of complex IIa. A20 is also under NF-κB transcriptional control, adding another layer of complexity to TNF/TNFR1 signalling crosstalk.

The ZnF7 domain of A20 binds to M1-linked ubiquitin chains and is required for recruitment of A20 to complex I [166, 167]. ZnF7-mutant mice develop arthritis [168], supporting a ZnF7-dependent role for A20 in regulating TNF/TNFR1 signalling. Binding of ZnF7 to M1-linked ubiquitin chains has been shown to protect them from degradation by DUB enzymes such as CYLD [96, 166]. CYLD (another NF-κB target gene) has been proposed to remove M1-linked ubiquitin chains from complex I components to destabilise the complex and promote a switch towards the formation of complex II [96, 166]. In protecting M1-linked chains from degradation, A20 suppresses TNF-induced cell death by stabilising complex I. Antagonising interactions between A20 and CYLD may provide a mechanism for regulating the interplay between complex I and complex II-mediated signalling pathways (Fig. 3). ZnF7-mediated recruitment of A20 to complex I also inhibits downstream activation of the IKK complex and thus negatively regulates pro-inflammatory signalling [169].

Global deficiency in RIPK3 significantly restores the survival of A20-deficient mice [170, 171], suggesting that A20 protects against complex II-induced necroptosis. In contrast, MLKL deficiency fails to rescue the early lethality of A20-deficient mice [171], further complicating the role of A20 in TNF/TNFR1 signalling. Although A20’s exact role in TNF/TNFR1 signalling remains unclear, it has a clear influence on the PTM profile of TNF/TNFR1 complex components. For instance, A20’s control over the level and type of ubiquitination on RIPK1 represents a crucial element for determining cell fate at the intersection of TNF/TNFR1 complex I and complex II signalling.

### Heterogeneity in TNF/TNFR1 signalling

Heterogeneity represents an inherent characteristic of cell populations and plays an important role in many regulatory processes. Cell-to-cell variability in TNF/TNFR1 signalling can be attributed to multiple mechanisms, including the dynamics of TNF secretion, receptor binding, signalling pathway interactions and gene expression [23, 32, 172]. Stochastic variation in protein-protein interactions between TNF and TNFR1 can have a significant impact on downstream signalling pathway activation. A minimum of two TNFR1-TNF contact points is required to activate the JNK/p38/NF-κB pathways [16]. Binding to fewer sites is sufficient to activate NF-κB but not JNK and p38 [16]. Within a population, TNFR1-TNF interactions could facilitate the activation of JNK/p38/NF-κB in some cells, whilst only NF-κB is activated in others. A further level of heterogeneity arises through the opposing negative and positive feedback loops that drive NF-κB inhibition (IκBα and A20) and TNF amplification (pulsatile secretion). Heterogenous NF-κB activation dynamics can produce diverse gene expression profiles [23, 28–30]. The dynamic profile of NF-κB could significantly influence the sensitivity of a cell to apoptosis by controlling the level of anti-apoptotic gene expression.

TNF/TNFR1 signalling cascades are inherently noisy due to stochastic fluctuations in genetic circuits [17]. This noise arises from variations in transcription and translation levels between cells, leading to differences in the expression of signalling components [173, 174]. This could also influence the composition of signalling complexes. For example, a reduction in cIAP1/2 expression promotes the formation of complex II [145]. Cell-to-cell variability can be observed in the kinetics of signalling reactions [175, 176]. Dynamic signalling events are significantly influenced by fundamental physical processes [177], such as cell cycle phase [178], growth rate [179], and the intrinsic promiscuity of protein-protein interactions [17]. Each of these dynamic signalling events must be properly integrated to determine the appropriate response of a cell to TNF, as apoptotic decision-making has a significant impact on both the individual cell and wider population. Cells respond heterogeneously to drugs that promote TNF-induced cell death by undergoing apoptosis at different time points. Some cells may also be resistant to treatment, leading to fractional killing [40, 180, 181]. Heterogeneity in TNF/TNFR1 signalling therefore likely provides a built-in mechanism to increase the survival probability of cell populations when exposed to an apoptotic-inducing stress.

### Elucidating the mechanisms driving heterogenous responses to TNF: the advantages and disadvantages of live-cell imaging

Conventional bulk-cell experimental techniques fail to truly capture cellular heterogeneity, as they provide average measurements across the entire cell population. Instead, methods capable of extracting information from individual cells within the population must be employed. Various aspects of cellular heterogeneity can be assessed using techniques such as scRNA-seq for gene expression [182], scATAC-seq for DNA accessibility [183, 184], scChIP–seq for histone modifications [185], and scBS-seq for DNA methylation [186]. Flow cytometry also enables single-cell analysis of phenotypes, including cell viability, surface marker expression and cell cycle phase [187]. These methods are all endpoint assays, meaning that cell behaviour cannot be continuously tracked over time within the same sample. This can be challenging for capturing transient signalling events, as it is difficult to treat and prepare samples within short timeframes. Since many cellular processes are dynamic, their investigation requires real-time non-invasive analysis of single cells. Non-invasive live-cell imaging has become the technology of choice to understand heterogeneous and dynamic processes. Ideally, this requires tools to study single molecule interactions, protein translocation and real-time analysis of gene expression [188]. Live-cell imaging of fluorescent-fusion proteins (FFP) has had a particularly important role, for example in the elucidation of NF-κB dynamics [23] (discussed in the next section).

Single-cell resolution fluorescence live-cell imaging techniques have been utilised to investigate heterogeneity in various cellular processes [23, 189–191]. FFPs have been generated to track the dynamics, localisation, and expression of proteins of interest [192, 193]. These widely used approaches for tracking protein localisation require the FFP to be expressed in the cell type of interest. Importantly, the FFP and its expression level must not interfere with protein function. It is important to check whether a C- or N-terminal fusion is optimal. Remarkably, fluorescent proteins have been found to often take on the stability of the protein they are fused to, as exemplified in the case of rapid signal-dependent degradation of enhanced green fluorescent protein (eGFP)-IκBα [23]. Various techniques have also emerged to support tracking of RNA molecules in cells to study transcription, translation, and RNA localisation within cells. A set of emerging techniques include, but are not limited to, the use of bacteriophage MS2 coat protein system [194–197], fluorogenic RNAs [198–200], and several RNA-targeting CRISPR-Cas systems [201–203]. Fluorophore-labelled probes such as Annexin V, propidium iodide and caspase-cleavable DEVD have also been used in a fluorescence live-cell imaging context to study cell death kinetics in response to specific reagents [204–206]. These fluorescent probes can also be utilised in flow cytometry assays to study cell death kinetics in a similar fashion [207].

Combining fluorescence live-cell microscopy with techniques such as immunofluorescence and immunohistochemistry can help to identify issues of perturbation of normal function. Fixing cells and staining for the endogenous protein of interest can confirm whether the same phenotype is identified compared to live-cell imaging experiments. Detection of fluorescent markers during live-cell imaging requires excitation of the fluorophore using a specific wavelength of light, and detection of light emitted at a longer wavelength [208]. Maintaining cellular health in a homoeostatic environment is a crucial component of fluorescence live-cell imaging [209] This requires ensuring constant temperature, humidity, pH, and osmolality. Excitation of the fluorophore should also be kept to a minimum to avoid oxidative stress and photobleaching. In many cases it is useful to use a DNA staining fluorescent dye to mark the nucleus, but care must be taken. For example, Hoechst 33342 is a popular DNA-staining dye but this can induce apoptosis due to phototoxicity from repeated excitation [210].

### Example of the use of live-cell imaging to investigate NF-kB dynamics

**Fig. 4:**
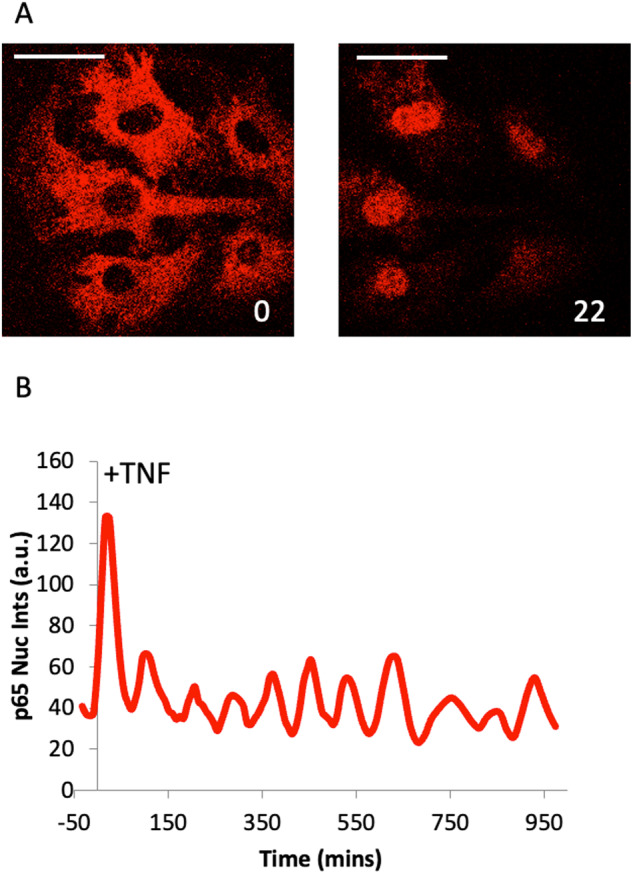
NF-κB oscillations in single cells. Mouse ear fibroblasts from an eGFP-RelA Bacterial Artificial Chromosome transgenic line were imaged on a Zeiss LSM780 confocal microscope every 2 min for 16 h following 10 ng/ml TNF stimulation. **A** Images at 0 min and 22 min after TNF stimulation (scale bar 10 microns) (**B**) Analysis of nuclear fluorescence in an example single-cell over time.

Initial studies quantified NF-κB dynamics by transiently transfecting plasmids expressing RelA-FFPs into cells [23]. Following TNF treatment, RelA translocation between the nucleus and the cytoplasm could be visualised in real-time and quantified by calculating the nuclear-cytoplasmic (N-C) ratio. More recently, stable RelA-FFP cell lines have been established using lentivirus [211, 212], Bacterial Artificial Chromosome-mediated expression [159, 213] (Fig. 4), and CRISPR/Cas9-mediated RelA-FFP knock-in [24, 214]. Stable RelA FFP lines offer the advantage of relatively uniform RelA-FFP expression levels [215]. Isogenic knock-in cell lines have the added benefit of enabling RelA-FFP expression from the endogenous RelA promoter, thus avoiding any behavioural artifacts associated with RelA overexpression.

GFP-RelA knock-in mice have been generated to study NF-κB dynamics [216]. Homozygous GFP-RelA mice can be used to derive various primary cell types, such as macrophages and endothelial cells. Employing fluorescence live-cell imaging to measure the activation dynamics of NF-κB in different primary cells derived from the same source could address cell-specific mechanisms controlling NF-κB activity [216]. Additionally, RelA N-C shuttling can be measured in vivo within individual cells of live GFP-RelA mice using intravital fluorescence microscopy [217]. Conducting experiments in live mice enables the continuous tracking of physiological changes over an extended period within the same research subject. This is particularly useful for studying cellular responses to TNF, as inflammation and cell death can manifest over a range of timescales, spanning from hours to years, depending on whether the response is acute or chronic [218, 219]. This approach can also be utilised to conduct experiments such as lineage tracing, allowing for the constant monitoring of cell-fate decisions over time [220, 221].

Utilising fluorescence live-cell imaging to measure NF-κB N-C shuttling revealed that the duration of TNF exposure influences NF-κB activation dynamics [23, 31]. Interestingly, Lee et al. [222] demonstrated that short pulses of TNF (1 min) are more effective at inducing apoptosis in HeLa (human cervical carcinoma) cells than longer pulses. Prolonged exposure to TNF increases the duration of NF-κB nuclear occupancy and thus increases the induction of NF-κB target genes [222]. Given that TNF/TNFR1 signalling through complex I inhibits complex II [100–103], and multiple NF-κB target genes protect against apoptosis [96, 138, 148, 149], sustained NF-κB activation caused by longer TNF pulses maintains the inhibition of complex II and thus reduces cell death [222, 223]. This finding underscores the importance of TNF/TNFR1 complex I and complex II signalling crosstalk in controlling cell-fate decisions in response to TNF. The duration of TNF exposure also provides additional opportunities for cell-to-cell variability, as a short pulse of TNF may be sufficient to induce apoptosis in some cells but not others. A further study utilised fluorescence live-cell microscopy alongside mathematical modelling to establish a connection between NF-κB dynamics and necroptosis in response to TNF [224]. A20 was identified as a key regulator in controlling the interplay between complex I-mediated NF-κB signalling and complex IIb-mediated necroptotic signalling in single cells. The decision for a cell to undergo necroptosis is proposed to be controlled by A20, which forms an incoherent feedforward loop during NF-κB signalling to protect a fraction of cells from transient TNF doses but renders them sensitive to long-term TNF exposure.

### Overcoming cellular heterogeneity in the treatment of cancer

Epigenetic variability within an isogenic cell population may be evolutionarily advantageous, as diverse responses to harmful stimuli increase the probability that a subpopulation of cells can survive [225]. Whilst the response of individual cells may differ due to both regulated and stochastic variations in cellular processes, robust phenotypes can be observed at the population level [32, 226]. Applied to the TNF/TNFR1 signalling pathway, cell-to-cell variability could ensure that TNF/TNFR1-disrupting pathogens do not fully eradicate the cell population by inducing apoptosis in all cells. Thus, cellular heterogeneity may provide a built-in safeguarding mechanism to ensure the continued survival of a cell population. Oyler-Yaniv et al. [227] developed the idea that TNF regulates a trade-off between cell death decision speed and accuracy in response to infection. Whilst infected cells die faster in the presence of TNF, this comes at the expense of increased death of uninfected bystander cells. The precise control of this trade-off in individual cells, which is likely regulated by TNF/TNFR1 signalling pathway crosstalk, is essential to restrict the spread of infection throughout the entire cell population.

Although epigenetic variability is a useful survival strategy for an isogenic cell population, it poses a significant challenge to the successful treatment of cancer. Intra-tumour heterogeneity (ITH) describes the existence of subpopulations within a tumour that exhibit distinct genetic, epigenetic, and phenotypic characteristics [228]. In the same way that heterogenous responses to TNF/TNFR1-disrupting pathogens increase the probability of survival for isogenic cell populations, ITH maximises the fitness of cancer cell populations in dynamic tumour environments [229]. There are at least three mechanisms driving ITH in cancer [1]: genetic heterogeneity, wherein cancer cells stochastically accumulate mutations through genomic instability, leading to the emergence of tumour subclones with distinct genotypes [2, 230]; non-genetic heterogeneity, resulting from variations in regulatory mechanisms, including epigenetic, posttranscriptional, and post-translational modifications [3, 229]; tumour microenvironmental (TME) heterogeneity, caused by region-specific selection pressures throughout different parts of the tumour [231]. These mechanisms are not mutually exclusive and work in concert, contributing to a complex system with multiple layers of heterogeneity [229]. In the context of TNF/TNFR1 signalling, ITH has the potential to create tumour subpopulations that exhibit increased resistance to TNF-induced cell death. Through modification of TNF/TNFR1 signalling components, cancer cells could modulate pathway crosstalk so that complex I-mediated cell survival is favoured over complex II-induced cell death, even in conditions where signalling through complex I is disrupted. These modifications could include changes to gene expression, PTM profile or genetic mutations.

‘Hot’ TMEs are characterised by high infiltration of immune cells, including cytotoxic lymphocytes (CL) and M1/M2-like tumour-associated macrophages [232, 233]. These immune cells release cytokines, giving rise to an inflammatory phenotype [234, 235]. The secretion of cytokines such as interferon γ, TNF, and TNF-related apoptosis-inducing ligand by CLs represents a key antitumour mechanism, as it induces proliferative arrest and/or apoptosis in target cells [236–242]. In immune hot TMEs with high concentrations of TNF, it would be advantageous for cancer cells to be more resistant to TNF-induced cell death. Indeed, Kearney et al. [240] revealed that tumour cells upregulate PD-L1 expression to suppress secretion of TNF and cell killing by CLs. Kearney et al. [241] further demonstrated that loss of the TNF/TNFR1 signalling components *Casp8* and *Tnfrsf1a* increases resistance to CD8 + T cell- and natural killer cell-mediated TNF-induced cell death, thus driving immune cell evasion in cancer.

The acquired resistance of cancer cells to TNF-induced cell death is currently a research area of interest, as modulators of the TNF/TNFR1 signalling pathway have the potential to sensitise resistant cancer cells to TNF [240, 242]. Recent studies have highlighted the importance of TNF-induced cell death in contributing to CAR T-cell cytotoxicity [243, 244]. Encouragingly, the antitumour activity of CAR T-cells can be significantly enhanced when cancer cells are made more susceptible to TNF-induced cell [243]. Table 1 provides an overview of the various mechanisms driving TNF-induced cell death that have currently been published.

**Table 1:** Mechanisms of acquired resistance to TNF-induced cell death in cancer.

Mechanism of resistance	References
Expression of *Rnf31* and *Vps4b*	[246, 247]
Expression of salt-inducible kinase 3	[248]
Expression of HOIP	[249]
Increased levels of autophagy	[250]
Loss of *Casp8*, *Tnfrsf1a* and *Ado* expression	[241]
Increased expression of PD-L1	[240]
Sub-lytic complement activation	[251]
Increased expression of Serpin B9	[252]
Expression of decoy receptors that sequester TNF	[253]
Increased expression of c-FLIP	[254, 255]

Establishing a mechanistic and quantitative understanding of the molecular processes underpinning heterogeneous responses to TNF could prove invaluable for increasing the efficacy of cancer treatments. Given that cellular heterogeneity poses a significant challenge to drugs that induce both intrinsic and extrinsic apoptosis, there is a need for techniques that offer real-time and dynamic insights into molecular processes in single cells. This review therefore advocates for the application of fluorescence live-cell imaging in the study of TNF/TNFR1 signalling, with a specific focus on how complex I and complex II signalling interactions govern cell-fate decisions. The generation of isogenic cell lines that endogenously express FFPs of TNF/TNFR1 signalling components would allow for quantitative and dynamic measurements of cellular responses to TNF in single cells. TNFR1, RIPK1 and A20 would be suitable candidates for this approach. Promisingly, recent studies have already begun utilising fluorescence live-cell microscopy to examine both intrinsic [245] and extrinsic [224] apoptosis, indicating a bright future for the field.

## Data Availability

All data generated or analysed during this study are included in this published article.
